# Recurrent Colorectal Liver Metastases: Upfront Local Treatment versus Neoadjuvant Systemic Therapy Followed by Local Treatment (COLLISION RELAPSE): Study Protocol of a Phase III Prospective Randomized Controlled Trial

**DOI:** 10.1007/s00270-023-03602-y

**Published:** 2023-11-09

**Authors:** Madelon Dijkstra, Babette I. Kuiper, Hannah H. Schulz, Susan van der Lei, Robbert S. Puijk, Danielle J. W. Vos, Florentine E. F. Timmer, Hester J. Scheffer, Tineke E. Buffart, M. Petrousjka van den Tol, Birgit I. Lissenberg-Witte, Rutger-Jan Swijnenburg, Kathelijn S. Versteeg, Martijn R. Meijerink, COLLISION Trial Group

**Affiliations:** 1 Department of Radiology and Nuclear Medicine, Amsterdam UMC, Location VUmc, Cancer Center Amsterdam, De Boelelaan 1117, 1081 HV Amsterdam, The Netherlands; 2 Department of Surgery, Amsterdam UMC, Location VUmc, Cancer Center Amsterdam, Amsterdam, The Netherlands; 3 Department of Radiology and Nuclear Medicine, Noordwest Ziekenhuisgroep, Alkmaar, the Netherlands; 4 Department of Medical Oncology, Amsterdam UMC, Location VUmc, Cancer Center Amsterdam, Amsterdam, The Netherlands; 5 Department of Surgery, Medical Center Leeuwarden, Leeuwarden, The Netherlands; 6 Department of Epidemiology and Data Science, Amsterdam University Medical Centers, Location VUmc, Vrije Universiteit Amsterdam, Amsterdam, The Netherlands

**Keywords:** Colorectal cancer, Colorectal liver metastases (CRLM), Neoadjuvant systemic therapy, Thermal ablation, Surgery, Randomized controlled trial (RCT)

## Abstract

**Purpose:**

The objective of the COLLISION RELAPSE trial is to prove or disprove superiority of neoadjuvant systemic therapy followed by repeat local treatment (either thermal ablation and/or surgical resection), compared to repeat local treatment alone, in patients with at least one recurrent locally treatable CRLM within one year and no extrahepatic disease.

**Methods:**

A total of 360 patients will be included in this phase III, multicentre randomized controlled trial. The primary endpoint is overall survival. Secondary endpoints are distant progression-free survival, local tumour progression-free survival analysed per patient and per tumour, systemic therapy-related toxicity, procedural morbidity and mortality, length of hospital stay, pain assessment and quality of life, cost-effectiveness ratio and quality-adjusted life years.

**Discussion:**

If the addition of neoadjuvant systemic therapy to repeat local treatment of CRLM proves to be superior compared to repeat local treatment alone, this may lead to a prolonged life expectancy and increased disease-free survival at the cost of possible systemic therapy-related side effects.

**Level of Evidence:**

Level 1, phase III randomized controlled trial.

**Trial Registration:**

NCT05861505. May 17, 2023.

**Graphical Abstract:**

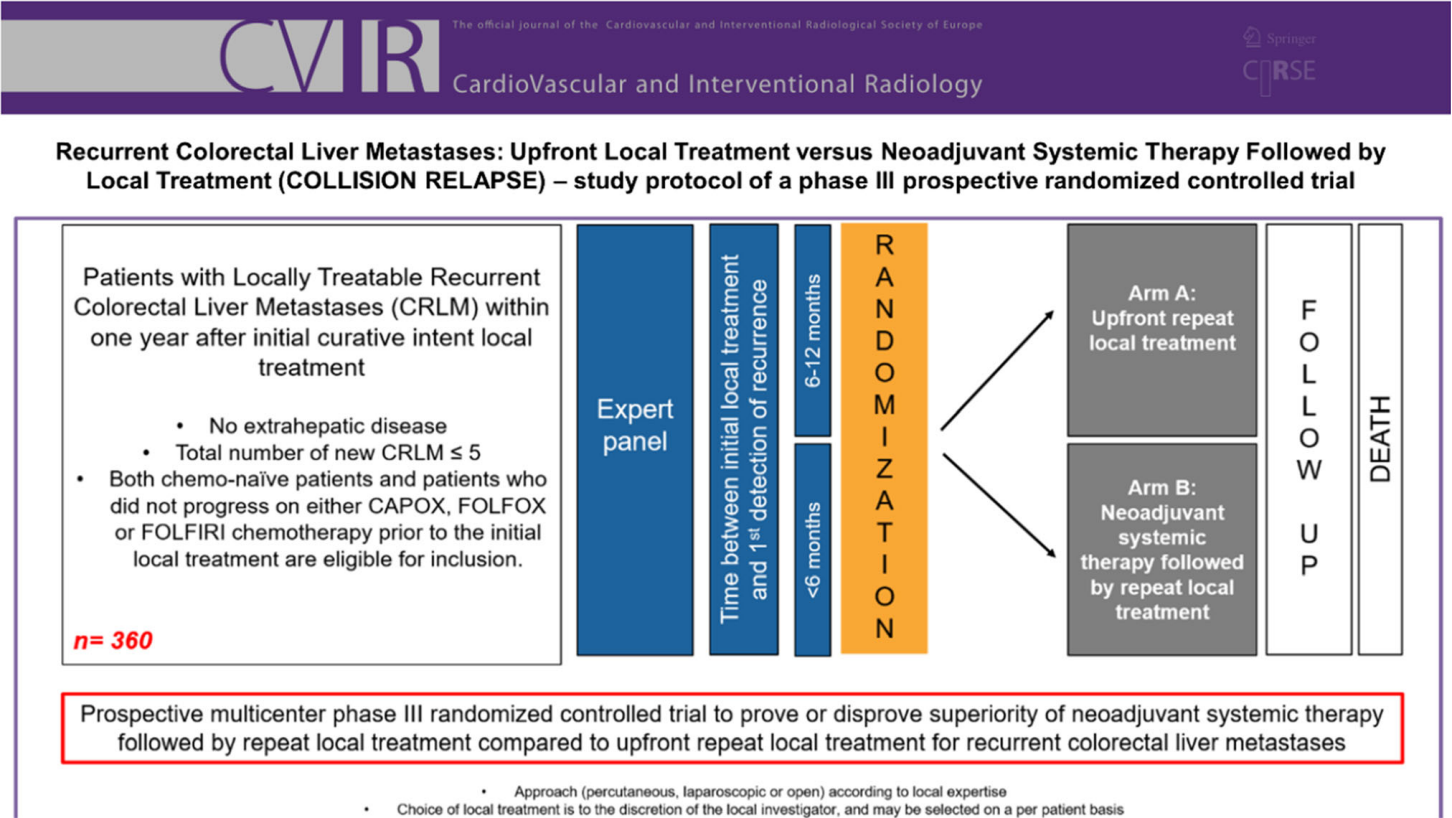

## Introduction

Colorectal cancer (CRC) is a common type of cancer, with an incidence of nearly two million new cases and a mortality rate of more than 900,000 deaths per year worldwide [1]. The prognosis largely depends on the presence of distant metastases. Up to 50% of patients with CRC develop colorectal liver metastases (CRLM) [2, 3]. Without any treatment, the 5-year overall survival (OS) rate is below 3% and when systemic therapy is administered, 5-year OS reaches 11% [4–6]. Local treatment comprising partial hepatectomy and/or thermal ablation offers potential cure, with 5-year OS rates of 44–58% [3, 7–9]. Despite complete tumour eradication, approximately 64–85% of patients develop new metastases after the first local treatment of CRLM [10, 11].

To treat intrahepatic recurrences, partial hepatectomy and/or thermal ablation are considered standard of care in current literature and international guidelines [12–16]. After upfront repeat local treatment, 5-year OS is 51% [17–20]. Early recurrent CRLM (≤ 12 months) are associated with poorer prognosis due to presumed worse tumour biology and the presence of intrahepatic micrometastases [21]. Heise et al. reported inferior disease-free survival (DFS) after repeat local treatment compared to the initial local treatment (*p* < 0.001) [22]. Therefore, neoadjuvant systemic therapy prior to repeat local treatment has been suggested to prolong both OS and DFS [23]. A recent pooled meta-analysis showed a trend towards improved survival with the addition of neoadjuvant systemic therapy to repeat local treatment [24]. Nevertheless, the side effects and toxicity of systemic therapy and impact on quality of life (QoL) should be carefully considered [25, 26].

The value of neoadjuvant systemic therapy prior to repeat local treatment in case of recurrent and locally treatable CRLM remains uncertain [24]. To assess the impact of neoadjuvant systemic therapy, we have designed a phase III randomized controlled trial (RCT) directly comparing upfront local treatment (control group) with neoadjuvant systemic therapy followed by local treatment (intervention group) in patients with recurrent CRLM within 12 months after initial local treatment.

## Design

### Design

The COLLISION RELAPSE trial is a phase III, multicentre randomized controlled trial initiated by the Amsterdam University Medical Centers (Amsterdam UMC), location VUmc in Amsterdam, the Netherlands. This study is endorsed by the Dutch Colorectal Cancer Group (DCCG) and by the Dutch Colorectal Cancer Foundation. Patients will be recruited in at least four centres in the Netherlands: Amsterdam UMC (location VUmc); Noordwest Ziekenhuisgroep Alkmaar; Leiden University Medical Center and Máxima Medisch Centrum, Veldhoven and Eindhoven. Additional Dutch and Belgian high volume liver centres are expected to contribute pending local approvals.

**Fig. 1:**
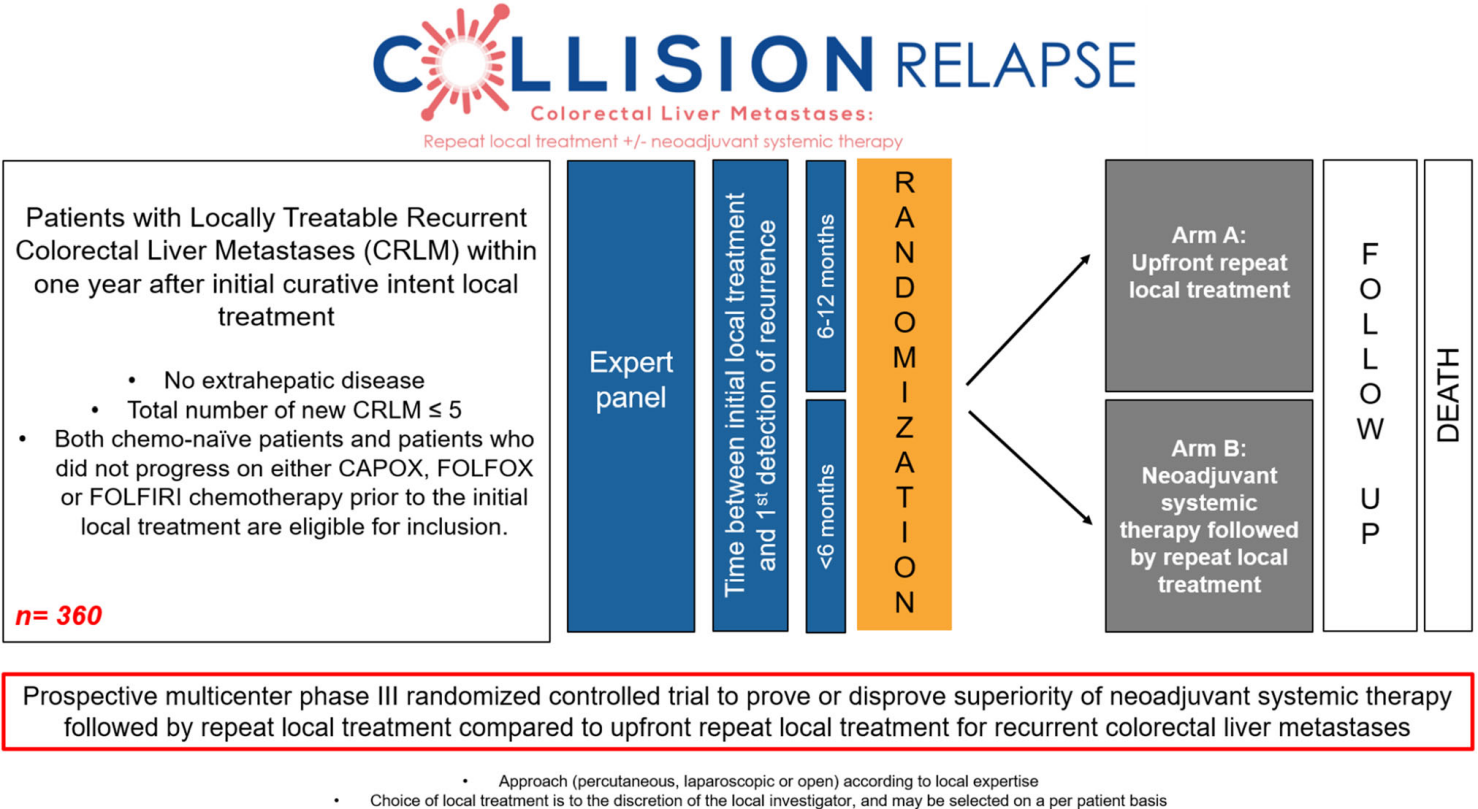
Flow chart of the study design

The trial is investigator-initiated, independent of industry and registered at clinicaltrials.gov under number NCT05861505. The protocol has been approved by the Amsterdam UMC, Medical Ethical Review Board (METc; no. 2022.0093—NL78220.029.21). The trial will be conducted in accordance with the Declaration of Helsinki and the guidelines for Good Clinical Practice (GCP). The flow chart of the study design is shown in Fig. 1. Inclusion, randomization and treatments started from April 2023 in four hospital centres.

### Inclusion Criteria

Patients with ≥ 1 locally treatable recurrent CRLM within 12 months following first local treatment, no microsatellite instability (MSI), no extrahepatic disease, and an Eastern Cooperative Oncology Group (ECOG) performance status of 0–2 and/or an American Society of Anesthesiologists (ASA) status of 1–3 are considered eligible (Table 1). Partial hepatectomy and/or thermal ablation is allowed with a maximum number of 5 recurrent CRLM.

**Table 1:** Inclusion and exclusion criteria

Inclusion criteria	Exclusion criteria
Age > 18 years	Extrahepatic disease
Performance status (ECOG 0–2 or ASA 1–3)	MSI/dMMR
Histological documentation of primary colorectal tumour (adenocarcinoma)	Radical local treatment unfeasible or unsafe (e.g. insufficient future liver volume)
Local treatment performed for initial CRLM	Compromised liver function (e.g. signs of portal hypertension, INR > 1.5 without use of anticoagulants, ascites)
New recurrence ≤ 12 months	Uncontrolled infections (> grade 2 NCI-CTC version 3.0)
≥ 1 locally treatable CRLM (resectable* and/or ablatable)	Pregnant or breast-feeding subjects
Total number of new CRLM ≤ 5	Immuno- or chemotherapy ≤ 6 weeks prior to the randomization
Chemo-naïve or history of response to CAPOX/FOLFOX/FOLRIRI	Severe allergy to contrast media not controlled with premedication
Life expectancy of at least 12 weeks	Substance abuse, medical, psychological or social conditions that may interfere with the subject’s participation in the study or evaluation of the study results
Adequate bone marrow, liver and renal function: Haemoglobin ≥ 5.6 mmol/L Absolute neutrophil count (ANC) ≥ 1,500/mm3 Platelet count ≥ 100*109/l Total bilirubin ≤ 1.5 times the upper limit of normal ALT and AST ≤ 2.5 × upper limit of normal (≤ 5 × upper limit of normal for subjects with liver involvement of their cancer) Albumin > 30 g/l Serum creatinine ≤ 1.5 × upper limit of normal or a MDRD ≥ 50 ml/min Prothrombin time or INR < 1.5 × ULN, unless coumarin derivates are used. Due to interactions with capecitabine, all patients using coumarin derivates will be treated with LMWH instead Activated partial thromboplastin time < 1.25 × ULN (therapeutic anticoagulation therapy is allowed if this treatment can be interrupted as judged by the treating physician)	
Written informed consent	

*ECOG* Eastern Cooperative Oncology Group, *ASA* American Society of Anesthesiologists, *MSI* Microsatellite instability, *dMMR* deficient mismatch repair

*Resection for resectable lesions considered possible by obtaining negative resection margins (R0) and preserving adequate liver reserve

### Exclusion Criteria

Patients with expert panel ineligibility prior to randomization will not be included. Recruited (included and randomized) patients are considered drop-outs when lost to follow-up or if patients actively decide to withdraw from the study at any time for any reason. Within the study protocol, crossover between treatment arms is not allowed. Patients in the intervention group will be excluded from the study if they refuse to start with neoadjuvant systemic therapy for any reason.

Regardless of the treatment arm, patients with previously undetected disease (e.g. detection of peritoneal metastases during surgery) or progressive remain study participants according to the intention-to-treat study design.

### Statistics

We hypothesize that neoadjuvant systemic therapy prior to repeat local treatment (either thermal ablation and/or surgical resection) is superior to direct local treatment for the selected patient groups in terms of the primary objective (overall survival). The Cox proportional hazards model (1-sided; superiority) is used for the sample size calculations. We consider OS improved if increased by 5% with 3-year follow-up with corresponding hazard ratio (HR) of 0.7 to represent the upper limit of superiority, based on guidelines provided by the Dutch Society of Medical Oncology [27]. The total number of events needed to estimate the HR of 0.7 with 80% at a significance level (alpha) of 0.05 is equal to 195. Following results of the LiverMetSurvey by Viganó et al. [28], 5-year probability of event is 55.9% (overall probability of event, pE = 0.559). Therefore, the calculated raw sample size is 348 (N_RS_). A 3% drop-out rate due to loss to follow-up or if patients actively decide to withdraw from the study at any time for any reason, is taken into account (N_DO_ = 12). A total number of 360 patients will be randomized (NR) into: arm A (control group) upfront repeat local treatment (*n* = 180) and arm B (intervention group) 12 weeks of neoadjuvant systemic therapy followed by repeat local treatment (*n* = 180).

All basic patient, procedure and tumour-related characteristics will be summarized and evaluated with standard descriptive statistics. Categorical variables will be tabulated with number of patients and analysed between arms using Fisher’s exact test and Pearson Chi square test. Continuous variables will be reported as means, standard deviations, medians and (interquartile) ranges. All *p* values below 0.05 will be considered significant.

The primary endpoint OS and secondary endpoints DPFS and LTPFS per patient and per tumour are defined as time-to-event from randomization and local treatment, respectively, and analysed using Kaplan–Meier curves with the log-rank test. In addition, Cox proportional hazards regression models are used to perform univariable and multivariable analysis on basic patient, procedure and tumour-related characteristics. Systemic therapy-related toxicity and procedural morbidity and mortality will be described using Common Terminology Criteria for Adverse Events 5.0 (CTCAE) [29] and compared between arms using Pearson Chi square test. The length of hospital stay will be assessed using Mann–Whitney U Test. Visual analogue scale questionnaires will be used to assess pain prior to, directly after and every three months after repeat local treatment and compared using linear mixed models. Quality of life questionnaires will be conducted prior to, and every three months after repeat local treatment, and will also be assessed prior to, during and after neoadjuvant systemic therapy. Differences between arms will be evaluated with linear mixed models. Incremental Cost-effectiveness Ratio (ICER) will be calculated with direct and indirect total cost care for both arms and used to perform a cost-utility analysis using Quality-adjusted Life Years (QALY) to calculate years of full health lived.

Statistical analyses will be conducted using SPSS® Version 28.0 (IBM®, Armonk, New York, USA) [30] and R version 4.0.3. (R Foundation, Vienna, Austria) [31]. The statistical plan is supported by a biostatistician (BLW).

### Study Cohort

All patients will be discussed in the local multidisciplinary liver tumour boards of the participating centre. Potential candidates will be registered and undergo a routine, national guideline-based pre-procedural work-up: contrast enhanced (ce)CT of the chest and abdomen, ceMRI including diffusion-weighted imaging (DWI), ^18^F-FDG PET-CT, anaesthetic review, baseline full blood examination, urea and electrolytes, renal function tests, liver enzymes and coagulation profile test.

The patient’s medical history and cross-sectional imaging will be reviewed for eligibility by an expert panel consisting of at least two abdominal radiologists, two interventional radiologists, two hepatobiliary surgeons and two medical oncologists. Patients will provide consent for multidisciplinary peer consultation. After referral of potential eligible patients from the tumour board, the expert panel then confirms inclusion and arranges randomization if these patients are indeed locally treatable (either by thermal ablation and/or surgical resection). A small proportion of patients are likely to be rejected by the expert panel. After confirmation by the expert panel, informed consent will be obtained and the patients will be randomized. In addition, cross-sectional imaging of all patients receiving neoadjuvant systemic therapy are re-reviewed by the expert panel after completing all cycles in this trial to detect possible changes in treatment plan and chemotherapeutical efficacy.

After written informed consent is obtained, patients will be included and randomized. Patients should be scheduled to start systemic therapy or undergo repeat local treatment within a period of six weeks following randomization. Choice of repeat local treatment is at the discretion of the local investigator in consultation with local multidisciplinary liver tumour boards of the participating centre. Upfront repeat partial hepatectomy and/or thermal ablation should be scheduled according to the national guidelines [32], at least 4 weeks and at most 12 weeks after the last cycle of systemic therapy.

### Randomization

Patients will be randomized into: arm A (upfront repeat local treatment) and arm B (neoadjuvant systemic therapy followed by repeat local treatment). Randomization is centralized and performed using Castor EDC (electronic data capture). Eligible patients will be stratified according to: the interval between initial local treatment and first detection of recurrent CRLM (≤ 6 months vs. 6–12 months), RAS mutations vs RAS wildtype, BRAF mutation vs BRAF wildtype, prognostic risk score (modified clinical risk score Brudvik et al. [33], low vs. high risk) and previous systemic therapy versus no previous systemic therapy.

### Partial Hepatectomy

Partial hepatectomy will be conducted under general anaesthesia during laparoscopy or open laparotomy, based on the judgement of the surgeon performing the procedure. The abdominal cavity will be explored in order to exclude extrahepatic tumour manifestations by an experienced hepatobiliary surgeon, i.e. a certified oncological surgeon with broad expertise (having performed and/or supervised > 100 liver tumour resection procedures).

The surgeon will remove all target lesions whether or not combined with thermal ablation performed by an interventional radiologist. The extent of the resection, the resection margins and the specific technique is at the discretion of the performing liver surgeon (but should have the intention and thus the preoperative estimation of a possible pathological R0 resection, while preserving a sufficient future liver remnant). Postoperative care will be on the recovery ward and subsequently on either the surgery ward or medium care whenever deemed necessary.

### Thermal Ablation

The interventional radiologist will ablate all target lesions whether or not combined with partial hepatectomy performed by a surgeon. Patients without contra-indications for a percutaneous approach will undergo percutaneous thermal ablation. Contra-indications for a percutaneous approach are proximity of critical structures. To avoid collateral damage to intestines a minimum distance to the stomach, small bowel and colon of 15 mm should be respected. Therefore, pneumo- or hydro-dissections are allowed. To assess technical efficacy, a ceCT or ceMRI should be performed at the time of the procedure. Patients with a contra-indication for a percutaneous approach will undergo open laparoscopic or ablation.

Thermal ablation (either radiofrequency ablation, RFA; or microwave ablation, MWA) is performed according to the CIRSE quality improvement guidelines with an intentional tumour-free ablation margin of at least 1 cm by an experienced operator, i.e. having performed and/or supervised > 100 thermal ablation procedures. The definition of a technically successful ablation is based upon the specific protocols established by the device manufacturers in combination with immediate post procedurally performed ultrasound in case of open approaches (fully hyperechoic ablation zone with an intentional margin of at least 1 cm) or imaging with confirmation software in case of percutaneous approaches. Necessity for re-ablations during the procedure (completion of the procedure) and/or needle repositioning will be judged by the performing interventional radiologist. Postoperative care will be on the recovery and subsequently on either the surgery ward or medium care whenever deemed necessary.

### Neoadjuvant Systemic Therapy

In case of randomization to arm B, patients receive maximum 12 weeks (4/6 cycles) of neoadjuvant systemic therapy. In case of delayed delivery of planned systemic therapy, the treatment will be maintained for a maximum duration of 12 weeks. Patients receive 4 cycles of CAPOX (capecitabine with oxaliplatin) or 6 cycles of FOLFOX/FOLFIRI (5-fluorouracil/leucovorin with either oxaliplatin or irinotecan), both with or without bevacizumab. The choice of agent is regardless of the location of the primary tumour, RAS mutation status, BRAF mutation status and previously received systemic therapy following primary tumour resection or previously received induction chemotherapy for initial downstaging of CRLM. The choice of treatment is at the discretion of the local medical oncologist.

A baseline ceCT or ^18^F-FDG-PET-CT will be performed no more than 28 days prior to the first dose of chemotherapeutic treatment. After 3 cycles of CAPOX or 4 cycles of FOLFOX/FOLFIRI (with or without bevacizumab), a follow-up ceCT will be acquired and response rates will be evaluated according to Response Evaluation Criteria in Solid Tumours (RECIST guideline, version 1.1) [34]. Patients who show clinical benefit, defined as stable disease or response to therapy, will be treated with additional 1 (CAPOX) or 2 (FOLFOX/FOLFIRI) cycle(s) of neoadjuvant systemic therapy without bevacizumab, followed by repeat local treatment. Patients with disease progression who, based on ceMRI, still qualify for repeat local treatment according to the expert panel, will receive repeat local treatment within 4–12 weeks. CRLMs in complete remission, confirmed by contrast enhanced magnetic resonance imaging (ceMRI), will not be locally treated but will be monitored using cross-sectional imaging. Patients with progressive disease will be treated according to best clinical practice, including all treatment modalities and remain in the trial group according to intention-to-treat. Supportive care for treatment-related symptoms will be offered as needed to all patients in this study.

### Follow-up

**Fig. 2:**
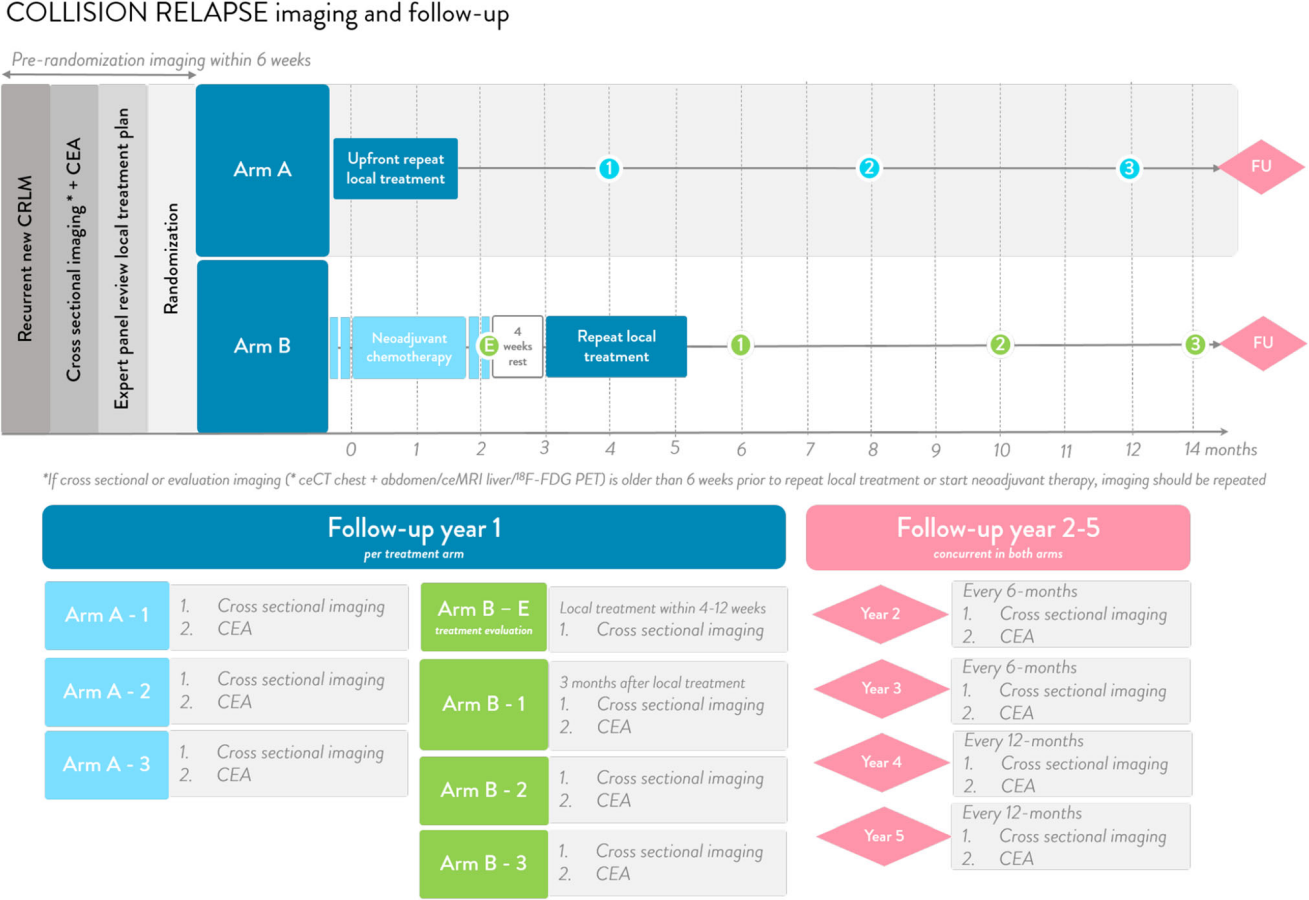
Follow-up scheme

The follow-up scheme is presented in Fig. 2 and based on (inter)national standards. Patients with disease progression will be treated according to best clinical practice, including all treatment modalities and remain in the trial group according to intention-to-treat.

### Outcome Measures

The main objective is to prove or disprove superiority of neoadjuvant systemic therapy followed by repeat local treatment compared to upfront repeat local treatment in patients with at least one locally treatable recurrent CRLM and no extrahepatic disease. The primary endpoint is overall survival (OS). Secondary endpoints are distant progression-free survival (DPFS), local tumour progression-free survival (LTPFS) per patient and per tumour, systemic therapy-related toxicity, procedural morbidity and mortality, length of hospital stay, pain assessment and QoL, cost-effectiveness ratio (ICER) and quality-adjusted life years (QALY).

### Data Monitoring

An independent monitor committee (Clinical Research Bureau) will maintain the quality of this investigator-initiated study according to GCP. Onsite monitoring including source data verification, to verify similarity between data on the case report form and source data, will be performed. In addition, all informed consent forms, inclusion and exclusion criteria and primary outcome OS will be confirmed for all participants.

### Serious Adverse Events

Lastly, all serious adverse events (SAE) will be reported to ToetsingOnline and the METc, after which the reports are checked on adequacy and compliance with legal rules and regulations. All SAE’s, both related and unrelated to the treatment will be reported within 15 days after notification, or within 7 days if the SAE is life-threatening or resulted in death.

## Discussion

Literature to support repeat local treatment of recurrent CRLM is well established. Multiple studies have shown a superior OS and DFS of repeat local treatment over palliative chemotherapy alone, resulting in a high number of long-term survivors [17–19]. Studying a selected group of patients receiving second local treatment, international large retrospective series show 5-year OS rates of nearly 51%, without compromising safety [17–19, 35]. International guidelines recommend either repeat partial hepatectomy and/or thermal ablation to treat recurrent CRLM, unless patients are not fit for local treatment or further treatment is not considered beneficial, then systemic therapy or palliative care is preferred [12–14, 36, 37]. For patients with recurrent disease after first local treatment, immediate repeated local treatment is considered to be the standard of care [12–15].

The EORTC 40983 trial by Nordlinger et al. and the JCOG 0603 trial by Kanemitsu et al. showed no benefit with the addition of (neo)adjuvant systemic therapy of resectable and/or ablatable disease after first local treatment of CRLM [38, 39]. Therefore, the role of systemic therapy remains reserved for limited purposes. For example, in to downstage CRLM to resectable and/or ablatable disease or to reduce procedural risks [40, 41]. However, the largest to date registry study (LiverMetSurvey) found an OS benefit favouring the use of neoadjuvant systemic therapy before repeat local treatment: 5-year OS: 61.5% versus 43.7% (HR = 0.529; *p* = 0.028) [28]. The authors advocate the use of neoadjuvant systemic therapy to adequately select good candidates for repeat local treatment and to control rapidly progressive disease. A recent pooled meta-analysis showed a trend towards improved survival, but the results remain indecisive and no conclusions could be drawn to define the role of neoadjuvant systemic therapy in recurrent CRLM [24].

Furthermore, recurrent disease is associated with micrometastatic disease and dormant cancer cells, which are not addressed by repeat local treatment alone [21]. This potentially indicates a higher risk profile, where providing aggressive systemic as well as local treatment is suggested[42, 43]. Moreover, the use of neoadjuvant systemic therapy may improve selection of patients eligible for repeat local treatment by adjusting treatment strategy to tumour biology and it may decrease risks of repeat local treatment [44–47]. If tumour shrinkage is observed during the administration of neoadjuvant systemic, studies suggested an increased rate of complete resection rates [44]. No (inter)national guideline organizations and scientific societies clearly discuss the position of neoadjuvant systemic therapy in recurrent CRLM.

Besides the potential benefits, the well-known risks and toxicities of systemic therapy should be taken into account [25, 26]. In addition, during the administration of neoadjuvant systematic therapy in recurrent CRLM, high rates of chemotherapeutic side effects and complications (46.7%) and lower QoL were found [48]. Other retrospective series did not report systematic therapy-related impact on repeat local treatment nor detected any increase in periprocedural complications or length of hospital [49, 50]. Neoadjuvant systemic therapy is specifically found to be safe if patients are not overtreated before surgical resection or thermal ablation [21]. No negative effect on periprocedural morbidity or liver function was found, when adhering to a maximum of 12 weeks of neoadjuvant systemic therapy [51–57].

In conclusion, neoadjuvant systemic therapy prior to repeat local treatment has been suggested to prolong survival, to eliminate micrometastatic disease, to eradicate dormant cancer cells in the liver, to decrease the risk of recurrences and to control rapidly progressive disease. However, the role of neoadjuvant systemic therapy in recurrent new and locally treatable CRLM remains uncertain. To assess the added value of neoadjuvant systemic therapy, we have designed a phase III randomized controlled trial (RCT) directly comparing upfront repeat local treatment (control) with neoadjuvant systemic therapy followed by repeat local treatment (intervention).

## Abbreviations

(S)AE: (Serious) Adverse Events
^18^F-FDG: [18F]-fluoro-2-deoxy-d-glucose
ASA: American Society of Anesthesiologists
BRAF: V-raf Murine Sarcoma Viral Oncogene Homolog B
CAPOX: Capecitabine with oxaliplatin
Ce: Contrast-enhancement
CEA: Carcinoembryonic Antigen
CRC: Colorectal Cancer
CRLM: Colorectal Liver Metastases
CT: Computed Tomography
CTCAE: Common Terminology Criteria for Adverse Events
DCCG: Dutch Colorectal Cancer Group
DPFS: Distant Progression-free Survival
DWI: Diffusion-weighted Imaging
ECOG: Eastern Cooperative Oncology Group
FOLFIRI: 5-Fluorouracil/leucovorin with irinotecan
FOLFOX: 5-Fluorouracil/leucovorin with oxaliplatin
GCP: Good Clinical Practice
ICER: Cost-Effectiveness Ratio
LTPFS: Local Tumour Progression-free Survival
METc: Medical Ethical Review Board
MRI: Magnetic Resonance Imaging
MSI: Microsatellite Instability
OS: Overall Survival
PET: Positron Emission Tomography
QALY: Quality-adjusted Life Years
QoL: Quality of Life
RAS: Rat Sarcoma Viral Oncogene Homolog
RCT: Randomized Controlled Trial

## Declarations

### Human and Animal Participants

All procedures performed in studies involving human participants will be in accordance with the ethical standards of the institutional and/or national research committee and with the 1964 Helsinki declaration and its later amendments or comparable ethical standards.
